# SELF-PERCEIVED COGNITIVE CHANGES IN OLDER BLACK ADULTS DURING COVID-19

**DOI:** 10.1093/geroni/igad104.2628

**Published:** 2023-12-21

**Authors:** Nancy Martens, Sophie Hanna, Hannah Adams, Loraine DiCerbo, Bruno Giordani, Voyko Kavcic

**Affiliations:** Wayne State University, Detroit, Michigan, United States; Wayne State University, Detroit, Michigan, United States; Wayne State University, Detroit, Michigan, United States; Wayne State University, Detroit, Michigan, United States; University of Michigan, Ann Arbor, Michigan, United States; Wayne State University, Detroit, Michigan, United States

## Abstract

The COVID-19 pandemic has had myriad effects on health and well-being. Contracting the COVID-19 virus and concerns of social and physical consequences during pandemic-related restrictions have been proven to negatively impact health. To better understand possible effects of COVID-19 on Black community-dwelling older adults’ perceived cognitive changes, we conducted a pilot study with 48 community-dwelling African Americans with measures obtained over 24 months from 10/2020 to 10/2022. Participants were recruited from the Wayne State University Institute of Gerontology Healthier Black Elders Center, and Michigan Alzheimer’s Disease Research Center, and the general Detroit Area (Mage=73.07, Range=65-87 years). The Cognitive Change Index (CCI) was used as a self-report measure of perceived change over the last five years. The COVID Impact Survey (CIS) was used to assess the burden of COVID-19 on daily living. To evaluate emotional-being, the Perceived Stress Scale (PSS responses referred to the last month) and the Geriatric Depression Scale (GDS, within the last week) were administered. Stepwise regression models showed CIS outcome measures did not predict CCI. GDS outcome measures predicted CCI to some degree (R2adj = .15, p = .008), whereas PSS was the strongest predictor (R2adj = .50, p < .001). Results suggest that for older community-dwelling African Americans, past COVID-19 pandemic experiences did not affect their self-evaluated cognitive changes. Higher levels of self-perceived stress within the last month best predicted their subjective cognitive decline. Future research should involve interventions focused on stress reduction, and clinicians should consider stress level when diagnosing cognitive status in older adults.

